# NcRNA-mediated upregulation of CAMK2N1 is associated with poor prognosis and tumor immune infiltration of gastric cancer

**DOI:** 10.3389/fgene.2022.888672

**Published:** 2022-08-25

**Authors:** Kaipeng Peng, Xiangqing Ren, Qian Ren

**Affiliations:** ^1^ The First Clinical Medical College, Lanzhou University, Lanzhou, China; ^2^ Department of Gastroenterology, The First Hospital of Lanzhou University, Lanzhou, China; ^3^ Key Laboratory for Gastrointestinal Diseases of Gansu Province, The First Hospital of Lanzhou University, Lanzhou, China

**Keywords:** CAMK2N1, gastric cancer, tumor immune infiltration, prognosis, noncoding RNA

## Abstract

Gastric cancer (GC) is still notorious for its poor prognosis and aggressive characteristics. Though great developments have been made in diagnosis and therapy for GC, the prognosis of patient is still perishing. In this study, differentially expressed genes (DEGs) in GC were first screened using three Gene Expression Omnibus (GEO) datasets (GSE13911, GSE29998, and GSE26899). Second, The Cancer Genome Atlas (TCGA) and Genotype-Tissue Expression (GTEx) data were used to validate expression of these DEGs and perform survival analysis. We selected seven candidate genes (CAMK2N1, OLFML2B, AKR7A3, CYP4X1, FMO5, MT1H, and MT1X) to carry out the next analysis. To construct the ceRNA network, we screened the most potential upstream ncRNAs of the candidate genes. A series of bioinformatics analyses, including expression analysis, correlation analysis, and survival analysis, revealed that the SNHG10–hsa-miR-378a-3p might be the most potential regulatory axis in GC. Then, the expression of CAMK2N1, miR-378a-3p, and SNHG10 was verified in GC cell lines (GES-1, MGC-803, BGC-823, HGC-27, MKN-45, and AGS) by qRT-PCR and Western blotting. We found that SNHG10 and CAMK2N1 were highly expressed in gastric cancer lines, and the miR-378a-3p was lowly expressed in BGC-823, HGC-27, and MKN-45. Furthermore, CAMK2N1 levels were significantly negatively associated with tumor immune cell infiltration, biomarkers of immune cells, and immune checkpoint expression. In summary, our results suggest that the ncRNA-mediated high expression of CAMK2N1 is associated with poor prognosis and tumor immune infiltration of GC.

## Introduction

Gastric cancer (GC) is one of the most common cancers in the world (Fu, 2015). The incidence of GC, especially in the Asian region, has observably increased over the past decades (Wang et al., 2015). In 2018, GC was the fifth incidence and third mortality among all cancers worldwide (Komatsu and Otsuji, 2019). This disease is a highly heterogeneous malignancy with different histological and molecular subtypes. Despite advances in surgery and neoadjuvant chemotherapy, the prognosis of patients with advanced gastric cancer remains poor, and the median overall survival (OS) was about 11 months, and the five-year survival rate was about 30% (Khorana et al., 2013; Digklia and Wagner, 2016; Smyth et al., 2016). Therefore, it is necessary to find effective therapeutic targets or seek valuable prognostic biomarkers in GC .

Non-coding RNAs (ncRNAs), which include miRNA, circRNA, and lncRNA, play a key role in human physiological and pathological activities (Anastasiadou et al., 2018; Kanwal and Lu, 2019). In recent years, ncRNAs have been the hotspot of human disease research (Esteller, 2011). NcRNAs have essential implications for human life activities; moreover, dysregulation of ncRNAs causes numerous disorders of human life activities, including cancer (Eddy, 2001). A competing endogenous RNA (ceRNA) hypothesis was proposed in 2011, which is a regulatory mechanism between mRNAs and ncRNAs (Salmena et al., 2011). The CeRNA mechanism assumes that competitively binding between lncRNAs, pseudogenes, circRNAs, and mRNAs to share miRNAs can regulate a series of biological functions (Karreth and Pandolfi, 2013). Increasing ncRNAs have been reported to influence tumor pathological processes (Wang et al., 2014a; Wang et al., 2017; Ding et al., 2019). In addition, ncRNAs have been found to have the value of potential diagnostic and prognostic biomarkers for cancer in blood (Qi et al., 2016; Zhang et al., 2017; Wei et al., 2019).

In this study, we constructed a ceRNA network linked to GC by a series of analytical processes. First, we identified differentially expressed genes (DEGs) associated with GC using three Gene Expression Omnibus (GEO) datasets (GSE13911, GSE29998, and GSE26899). Second, TCGA GC cohort was used to validate the expression of these DEGs and perform survival analysis. In addition, we excluded DEGs, which have been reported as prognostic biomarkers in GC. Subsequently, upstream miRNAs of the remaining DEGs were predicted using the starBase database. By expression correlation analysis and survival analysis, we identified the most potential miRNAs bound to CAMK2N1 and FOM5. Then, we identified the most potential lncRNA in the same way. Finally, we determined the association of CAMK2N1 expression with immune cell infiltration, biomarkers of immune cells, and immune checkpoints in GC. To confirm the reliability of the analysis results, we validated the expression of CAMK2N1 and ncRNAs in the human gastric cancer cell lines. To sum up, we found that ncRNA-related upregulation of CAMK2N1 correlates with poor prognosis and tumor immune infiltration of patients in GC.

## Materials and methods

### Microarray

The mRNA microarrays of GC were downloaded from the Xiantao database (https://www.xiantao.love/). We searched the GEO database with keywords such as “gastric cancer” and “mRNA.” GSE13911 (containing 37 tumor tissues and 32 normal tissues), GSE29998 (49 tumor tissues and 50 normal tissues), and GES26899 (95 tumor tissues and 13 normal tissues) were selected for the follow-up analysis of this study.

### Differential expression analysis of mRNAs

The Xiantao database (https://www.xiantao.love/) is an online analytical tool, which was used to perform differential expression analysis based on the GEO database. DEGs were screened out using adjusted *p*-value <0.05, |log2FC| > 1.

### GEPIA database analysis

GEPIA (http://gepia.cancer-pku.cn/index.html) is a novel tool for gene-expression profiling and interactive analyses in different types of cancer which is based on TCGA and Genotype-Tissue Expression (GTEx) data. We used this tool to determine DEG expression in GC. A *p*-value <0.05 was considered statistically significant.

### Kaplan–Meier plotter analysis

The Kaplan–Meier plotter (http://kmplot.com/analysis/) is an online database which can access the prognostic values of genes or microRNAs in more than 20 cancer types including GC. A log rank *p*-value <0.05 was considered statistically significant.

### starBase database analysis

starBase (http://starbase.sysu.edu.cn/) is a database for miRNA-related studies. starBase was introduced to perform expression correlation analysis for miRNA–DEGs, lncRNA–miRNA, and lncRNA–DEGs in GC. In addition, starBase was used to predict candidate miRNAs that could potentially bind to DEGs and lncRNAs that could bind to miRNAs. A *p*-value <0.05 was considered statistically significant.

### miRNet database analysis

The miRNet database (https://www.mirnet.ca/) is a web server for integrated data from several miRNA-linked databases and was used to predict the potential lncRNAs binding to miRNAs. Then, these lncRNAs were intersected with the lncRNAs obtained from the starBase database to acquire the most potential upstream lncRNAs of hsa-miR-378a-3p and hsa-miR-140-5p.

### TIMER database analysis

TIMER (https://cistrome.shinyapps.io/timer/) is an online tool for comprehensive relation analysis of tumor-infiltrating immune. TIMER was used to analyze the correlation of the CAMK2N1 expression level with the immune cell infiltration level or immune checkpoint expression level in GC. A *p*-value <0.05 was considered statistically significant.

### LinkedOmics database analysis

The LinkedOmics database (http://www.linkedomics.org/login.php) is a website for analyzing multi-omics data based on TCGA datasets. We obtained the differentially expressed genes related to CAMK2N1 in GC using the LinkFinder module, which were analyzed by the Pearson correlation coefficient and visualized by volcano plot and heat maps. To get descriptive information, the differentially expressed genes related to CAMK2N1 were annotated using Gene Ontology (GO) analysis and Kyoto Encyclopedia of Genes and Genomes (KEGG) pathway enrichment analysis.

### Cell lines

Normal human gastric mucosa epithelial cell line GES-1 and gastric cancer cell lines AGS, MGC-803, BGC-823, HGC-27, and MKN-45 were purchased from the Cell Bank of the Chinese Academy of Sciences (Shanghai, China). The cells were cultured in Dulbecco’s modified Eagle’s medium (Thermo Fisher Scientific, Waltham, United States) supplemented with 10% fetal bovine serum (Thermo Fisher Scientific, Waltham, United States), 100 U/ml penicillin, and 100 μg/ml streptomycin in a humidified atmosphere at 37 °C with 5% CO_2_.

### RNA extraction and qRT-PCR analysis

Total RNA from cells was extracted using TRIzol solution (Invitrogen, CA, United States). RNA was reverse-transcribed into complementary DNA (cDNA) by One-Step gDNA Removal and cDNA Synthesis SuperMix (TransGen Biotech, Beijing, China). qRT-PCR was conducted with Top Green qPCR SuperMix (TransGen Biotech, Beijing, China) at 94°C for 30 s, followed by 40 cycles at 94°C for 5 s and at 60°C for 30 s. Gene-specific qRT-PCR primers were purchased from Invitrogen (Shanghai, China). GAPDH or U6 was used as an internal control. The relative gene expression levels were calculated using the 2^−ΔΔCt^ method.

### Western blotting

Gastric cancer cells were lysed in RIPA buffer (Solarbio, Beijing, China) containing protease inhibitor for 30 min at 4 °C. The protein concentration was determined by the BCA protein assay kit (Thermo Fisher Scientific, MA, United States) according to the manufacturer’s instructions. The protein extracts were separated by 12% SDS-PAGE electrophoresis (30 μg/lane) and transferred to polyvinylidenedifluoride (PVDF) membranes (Millipore, MA, United States). The membranes were blocked with TBST containing 5% non-fat milk for 1 h and incubated overnight at 4 °C with primary antibodies against CAMK2N1 (1:1000, Invitrogen, MA, United States) and β-actin (1:1000, PTM BIO, Shanghai, China). The horseradish peroxidase (HRP)-conjugated secondary antibody (1:10,000, Invitrogen, MA, United States) was incubated at room temperature for 1 h. A chemiluminescence detection reagent (Thermo Fisher Scientific, MA, United States) was used to detect the signals.

### The primer sequence

Please refer to Supplementary Table S1 for the details.

### Statistical analysis

Statistical analysis was performed through GraphPad Prism (version 8, San Diego, CA). Student’s t-tests were utilized for the comparison of two groups. Differences were considered statistically significant when *p* < 0.05.

## Results

### Screening of candidate genes related to GC

**FIGURE 1:**
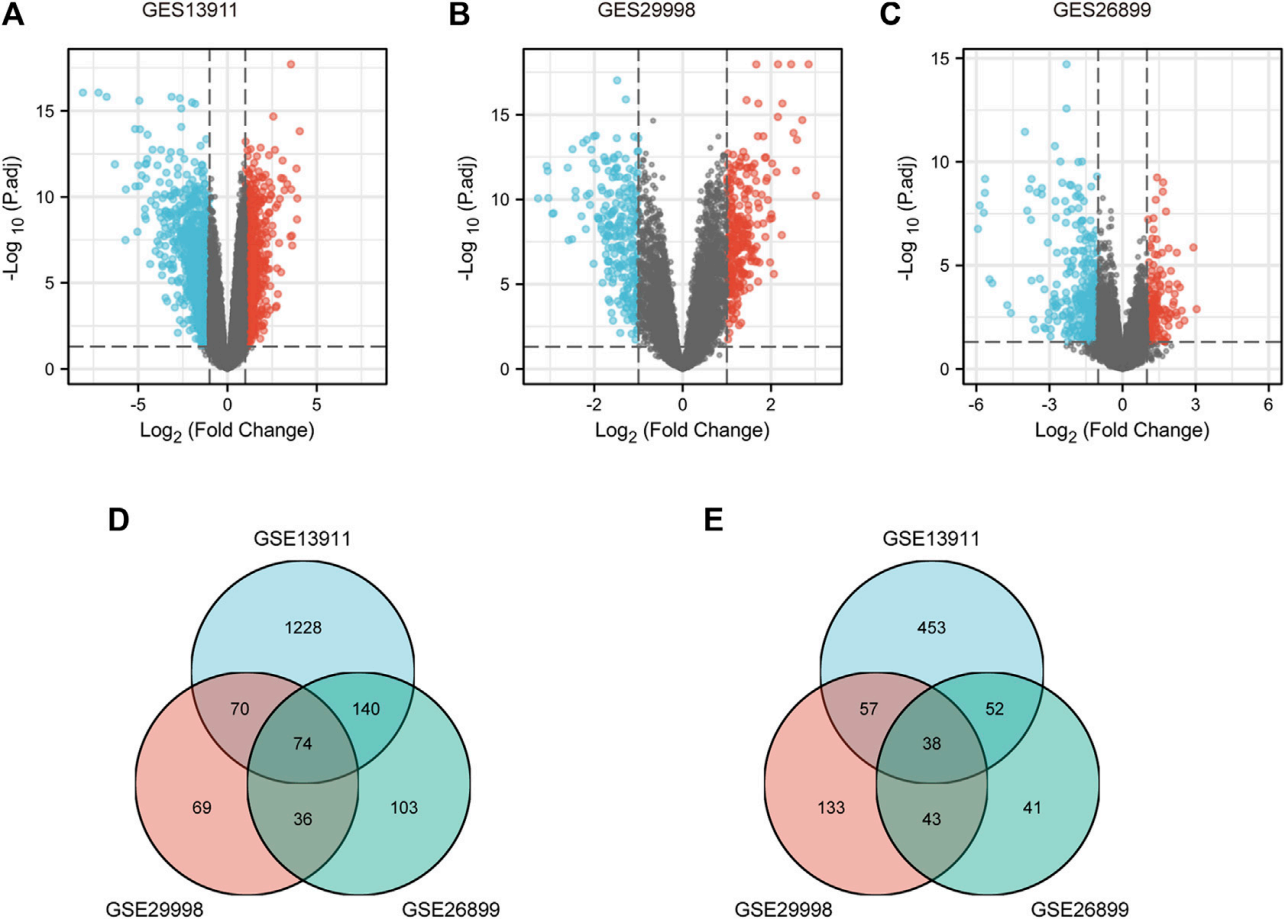
Identification of DEGs between gastric cancer and normal controls. **(A)** Volcano plots of gene expression profile data in GSE13911, containing 37 tumors tissues and 32 normal tissues. **(B)** Volcano plots of gene expression profile data in GSE29998, containing 49 tumors tissues and 50 normal tissues. **(C)** Volcano plots of gene expression profile data in GSE26899, containing 95 tumors tissues and 13 normal tissues. **(D)** Intersection of downregulated DEGs of GSE13911, GSE29998, and GSE26899. **(E)** Intersection of upregulated DEGs of GES19828, GSE13911, GSE29998, and GSE26899. DEG, differentially expressed gene.

To identify the key genes associated with GC, GES13911, GES29998, and GES26899 datasets were selected to perform differential expression analysis using the Xiantao database. As described in Materials and methods, the cases of GSE13911, GSE29998, and GES26899 are, respectively, divided into two groups: “normal” group and “tumor” group. Differentially expressed genes (DEGs) of three GEO datasets were discovered as shown in Figure 1A, Figure 1B, and Figure 1C, respectively. This study aims to find the most potential genes associated with GC. Therefore, we obtained the upregulated and downregulated DEGs that appeared in three datasets. As shown in Figures 1D,E, 38 upregulated DEGs and 74 downregulated DEGs were finally identified. Finally, the 112 DEGs were defined as candidate genes and processed for the subsequent analysis.

### Expression validation and survival analysis of candidate genes in GC

**FIGURE 2:**
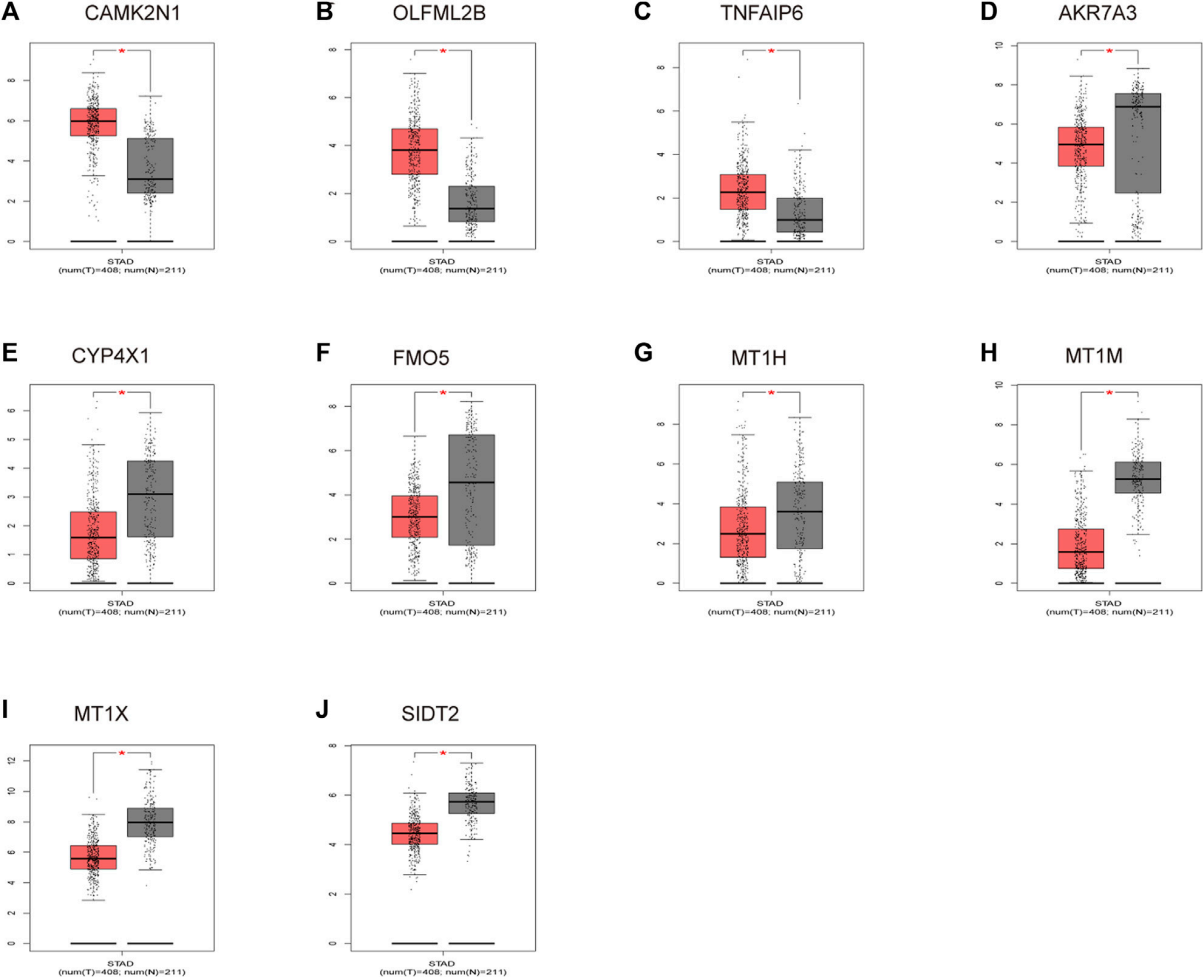
Expression levels of 10 key genes. **(A)** CAMK2N1. **(B)** OLFML2B. **(C)** TNFAIP6. **(D)** AKR7A3. **(E)** CYP4X1. **(F)** FMO5. **(G)** MT1H. **(H)** MT1M. **(I)** MT1X. **(J)** SIDT2. These genes were determined by the GEPIA database in gastric cancer. “*” represents a *p*-value < 0.05.

**FIGURE 3:**
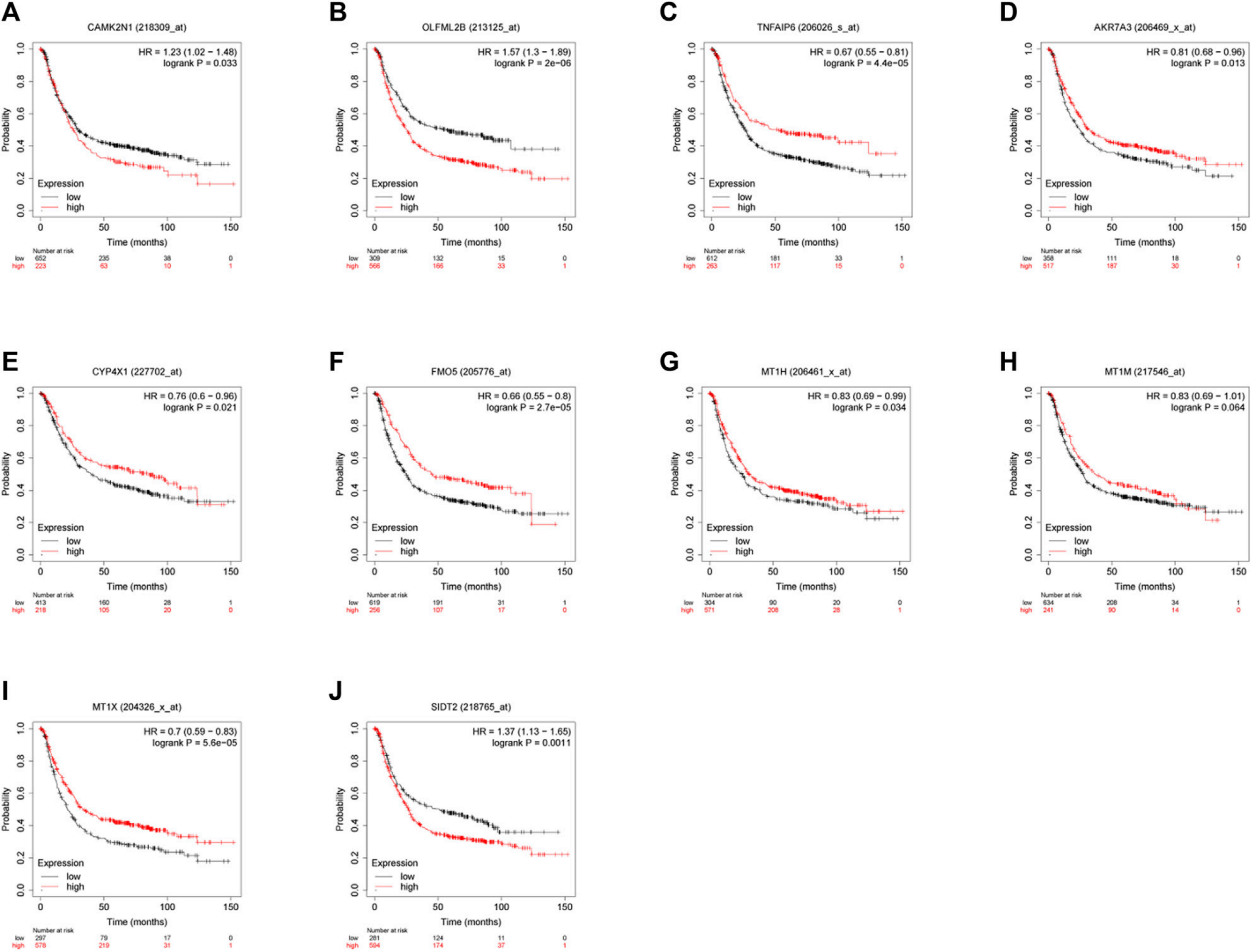
Prognostic values of 10 candidate genes in gastric cancer assessed using the Kaplan–Meier plotter database. **(A)** Prognostic value of CAMK2N1 in gastric cancer. **(B)** Prognostic value of OLFML2B in gastric cancer. **(C)** Prognostic value of TNFAIP6 in gastric cancer. **(D)** Prognostic value of AKR7A3 in gastric cancer. **(E)** Prognostic value of CYP4X1 in gastric cancer. **(F)** Prognostic value of FMO5 in gastric cancer. **(G)** Prognostic value of MT1H in gastric cancer. **(H)** Prognostic value of MT1M in gastric cancer. **(I)** Prognostic value of MT1X in gastric cancer. **(J)** Prognostic value of SIDT2 in gastric cancer. Log rank *p* < 0.05 was considered statistically significant.

**FIGURE 4:**
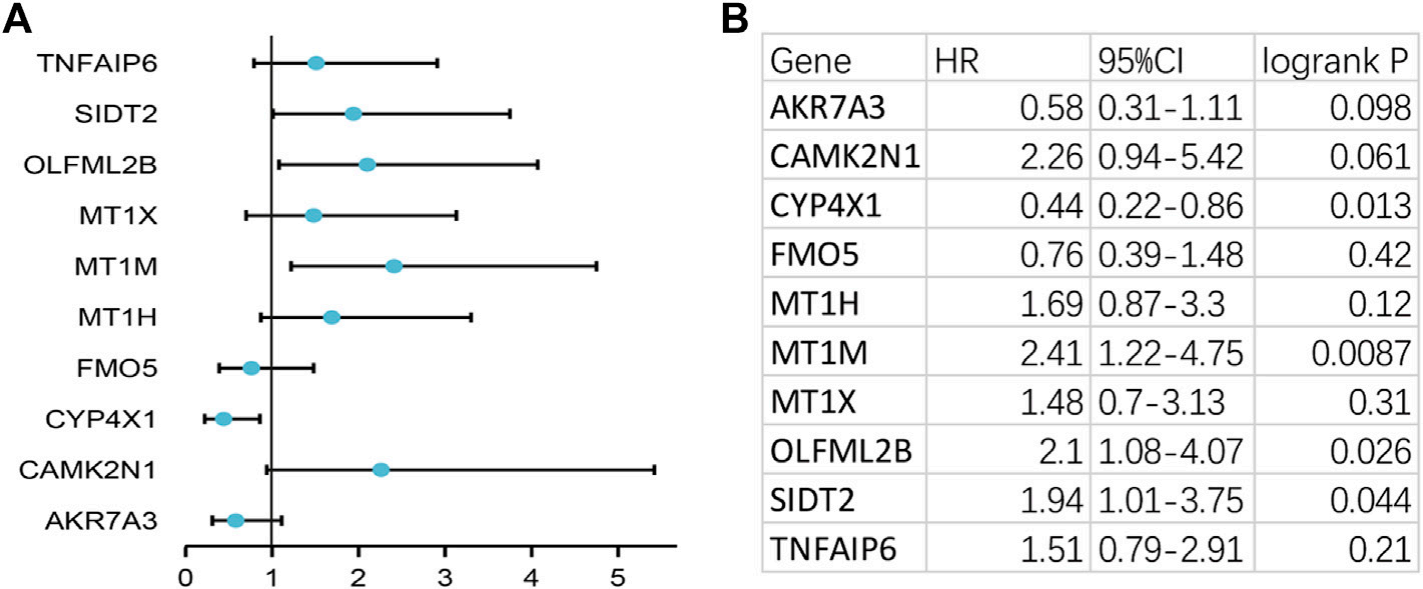
Prognostic values of 10 candidate genes in gastric cancer assessed using the Kaplan–Meier plotter database. **(A)** Prognostic values (relapse-free survival, RFS) of the 10 key genes in TCGA gastric cancer cohort. **(B)** Detailed information of **(A)**. Log rank *p* < 0.05 was considered statistically significant.

To verify the reliability of the analysis method, TCGA data and GTEx normal samples were used to validate the expression levels of 112 candidate genes by the GEPIA database. DEGs which had been reported as prognostic biomarkers in GC and were not in accordance with the GEO expression levels were excluded. As shown in Figure 2, among the 112 candidate genes, the expression of 10 genes (CAMK2N1, OLFML2B, TNFAIP6, AKR7A3, CYP4X1, FMO5, MT1H, MT1M, MT1X, and SIDT2) was selected. In the following survival analysis, we focused on the 10 key genes. First, TCGA GC cohort was used to assess the prognostic values (including OS and RFS) of the 10 candidate genes by the Kaplan–Meier plotter database. As shown in Figure 3, high expression of CAMK2N1, OLFML2B, and SIDT2 indicated a poor OS, and high expression of TNFAIP6, AKR7A3, CYP4X1, FMO5, MT1H, MT1M, and MT1X forecasted a better OS. But, the OS of MT1M was not statistically significant, while the OS of TNFAIP6 and SIDT2 were not in accordance with expression levels in tumor tissues. For RFS, high expression of OLFML2B, MT1M, and SIDT2 indicated a poor RFS of patients with GC, whereas gastric cancer patients with higher expression of CYP4X1 possessed better RFS from TCGA cohort (Figure 4). Therefore, we conducted the next analysis on CAMK2N1, OLFML2B, AKR7A3, CYP4X1, FMO5, MT1H, and MT1X (Table 1).

**TABLE 1:** Table 1 Expression change of 10 candidate genes in gastric cancer.

Gene name	Log2FC^a^	Log2FC^b^	Log2FC^c^
CAMK2N1	1.11	1.30	1.89
OLFM2B	1.04	2.25	1.20
TNFAIP6	1.49	1.50	1.05
AKR7A3	−1.76	−2.17	−1.70
CYP4X1	−1.63	−1.57	−1.33
FMO5	−1.71	−1.78	−1.10
MT1H	−1.45	−2.50	−2.12
MT1M	−2.75	−3.07	−1.80
MT1X	−1.15	−1.51	−1.01
SIDT2	−1.51	−1.29	−1.09

FC^a^ determined by GES13911.

FC^b^ determined by GES29998.

FC^c^ determined by GES26899.

### Prediction and analysis of upstream miRNAs of seven key genes

**FIGURE 5:**
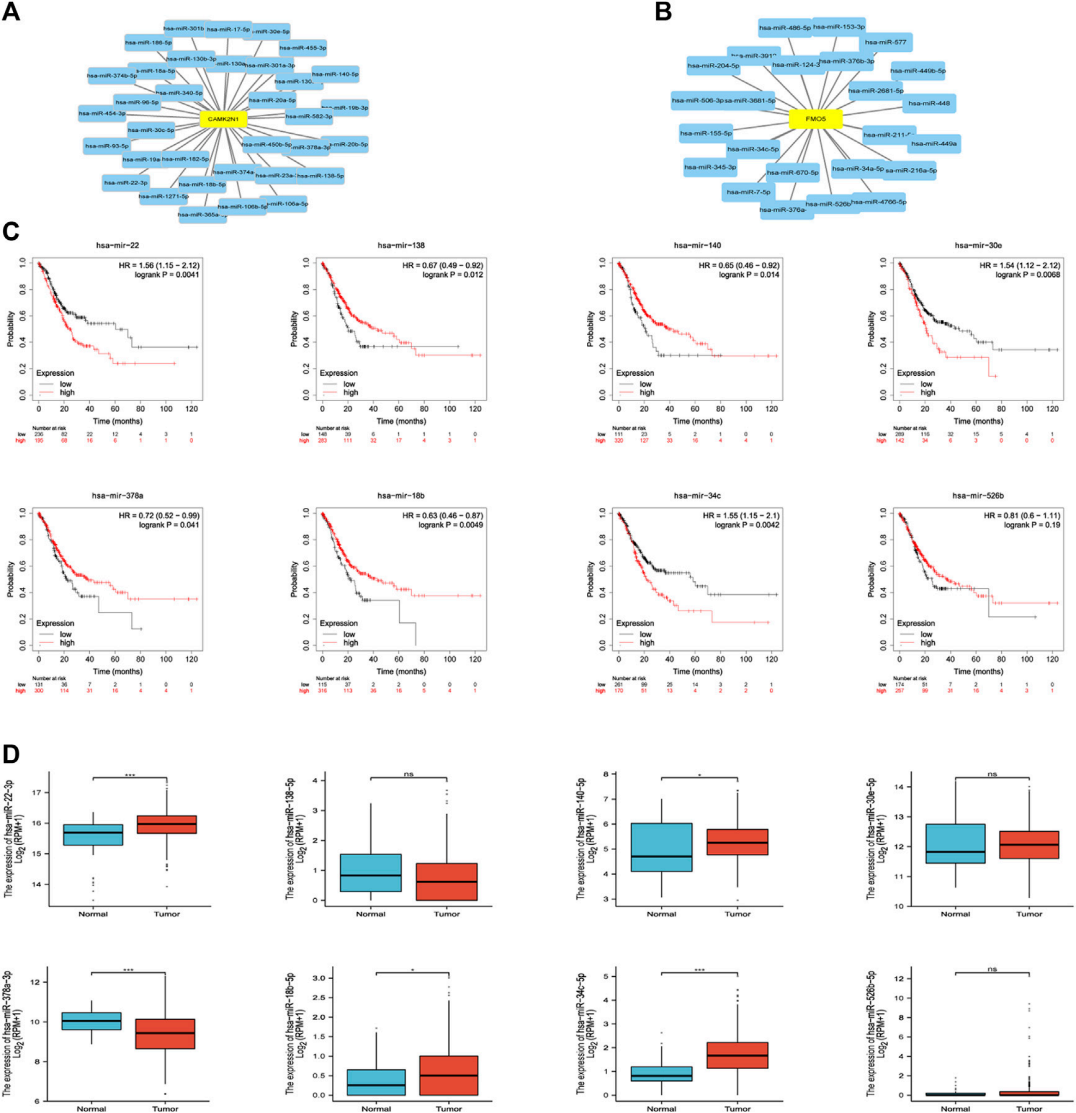
**(A)** miRNA–CAMK2N1 network established by Cytoscape software. **(B)** miRNA–FMO5 network established by Cytoscape software. **(C)** Prognostic values (overall survival, OS) of potential upstream miRNAs in TCGA gastric cancer cohort. **(D)** Expression of potential upstream miRNAs in TCGA gastric cancer cohort. “*” represents a *p*-value < 0.05; “***” represents a *p*-value < 0.01.

The regulation of gene expression by ncRNAs has been widely recognized. To ascertain whether key genes were regulated by some ncRNAs, we predicted upstream miRNAs that could potentially bind to candidate genes and verified the correlation between miRNAs and candidate genes. To improve visualization, a miRNA–mRNA regulatory network was established using Cytoscape software (Figures 5A,B). According to the mechanism of miRNA regulating the expression of target genes, there should be a negative correlation between miRNA and mRNA. As listed in Table 2, CAMK2N1 was significantly negatively correlated with hsa-miR-140-5p, hsa-miR-22-3p, hsa-miR-30e-5p, hsa-miR-138-5p, hsa-miR-18b-5p, and hsa-miR-378a-3p, and FMO5 was negatively correlated with hsa-miR-34c-5p and hsa-miR-526b-5p in GC. There were no statistical expression relationships or negative correlation between other mRNAs and the predicted miRNAs. Finally, the expression and prognostic value of candidate miRNAs in GC were determined by TCGA cohort. As presented in Figures 5C,D, hsa-miR-140-5p and hsa-miR-378a-3p were markedly downregulated in GC, and their upregulation was positively linked to patients’ prognosis. hsa-miR-34c-5p was upregulated in GC, and its downregulation was positively linked to patients’ prognosis. All these results suggest that hsa-miR-378a-3p–CAMK2N1 and hsa-miR-34c-5p–FMO5 could be the potential regulatory pathways.

**TABLE 2:** Prediction of miRNAs binding to CAMK2N1 and FMO5.

Gene name	miRNA name	Predicting program	Number	R	P
CAMK2N1	hsa-miR-17-5p	PITA, miRmap, miRanda, PicTar, and TargetScan	5	0.012	8.15E-01
CAMK2N1	hsa-miR-18a-5p	PITA, miRmap, microT, miRanda, PicTar, and TargetScan	6	−0.011	8.38E-01
CAMK2N1	hsa-miR-19a-3p	PITA, microT, and miRanda	3	−0.031	5.54E-01
CAMK2N1	hsa-miR-19b-3p	PITA, microT, and miRanda	3	0.02	7.03E-01
CAMK2N1	hsa-miR-20a-5p	PITA, miRmap, microT, miRanda, PicTar, and TargetScan	6	−0.009	8.64E-01
CAMK2N1	hsa-miR-22-3p	PITA, miRanda, PicTar, and TargetScan	4	0.146	4.74E-03
CAMK2N1	hsa-miR-23a-3p	PITA, miRanda, and TargetScan	3	0.029	5.74E-01
CAMK2N1	hsa-miR-93-5p	PITA, miRmap, miRanda, PicTar, and TargetScan	5	0.016	7.62E-01
CAMK2N1	hsa-miR-96-5p	PITA, miRmap, microT, miRanda, PicTar, and TargetScan	6	0.026	6.16E-01
CAMK2N1	hsa-miR-106a-5p	PITA, miRmap, microT, miRanda, PicTar, and TargetScan	6	−0.092	7.48E-02
CAMK2N1	hsa-miR-30c-5p	PITA, microT, miRanda, PicTar, and TargetScan	5	0.028	5.92E-01
CAMK2N1	hsa-miR-182-5p	PITA, microT, miRanda, PicTar, and TargetScan	5	0.013	8.04E-01
CAMK2N1	hsa-miR-138-5p	PITA, miRmap, microT, miRanda, PicTar, and TargetScan	6	−0.169	1.03E-03
CAMK2N1	hsa-miR-140-5p	PITA, miRmap, miRanda, PicTar, and TargetScan	5	−0.145	5.12E-03
CAMK2N1	hsa-miR-106b-5p	PITA, miRmap, microT, miRanda, PicTar, and TargetScan	6	0.04	4.38E-01
CAMK2N1	hsa-miR-30e-5p	PITA, microT, miRanda, and TargetScan	4	−0.185	3.42E-04
CAMK2N1	hsa-miR-378a-3p	PITA and miRanda	2	−0.122	1.83E-02
CAMK2N1	hsa-miR-18b-5p	PITA, miRmap, microT, miRanda, and TargetScan	5	−0.107	3.95E-02
CAMK2N1	hsa-miR-20b-5p	PITA, miRmap, miRanda, PicTar, and TargetScan	5	−0.05	3.38E-01
CAMK2N1	hsa-miR-455-3p	PITA, miRmap, microT, and TargetScan	4	−0.061	2.44E-01
CAMK2N1	hsa-miR-450b-5p	PITA, miRmap, and microT	3	−0.098	6.00E-02
CAMK2N1	hsa-miR-1271-5p	PITA, miRmap, microT, miRanda, and TargetScan	5	−0.092	7.68E-02
FMO5	hsa-miR-34a-5p	miRmap and miRanda	2	0.041	4.26E-01
FMO5	hsa-miR-153-3p	miRmap and miRanda	2	0.284	2.53E-08
FMO5	hsa-miR-34c-5p	miRmap and miRanda	2	−0.103	4.61E-02
FMO5	hsa-miR-448	miRmap and miRanda	2	−0.003	9.55E-01
FMO5	hsa-miR-449a	miRmap and miRanda	2	0.037	4.77E-01
FMO5	hsa-miR-526b-5p	miRmap and microT	2	−-0.148	4.24E-03
FMO5	hsa-miR-449b-5p	miRmap and miRanda	2	0.058	2.68E-01

### Prediction and analysis of upstream lncRNAs of candidate miRNAs

**FIGURE 6:**
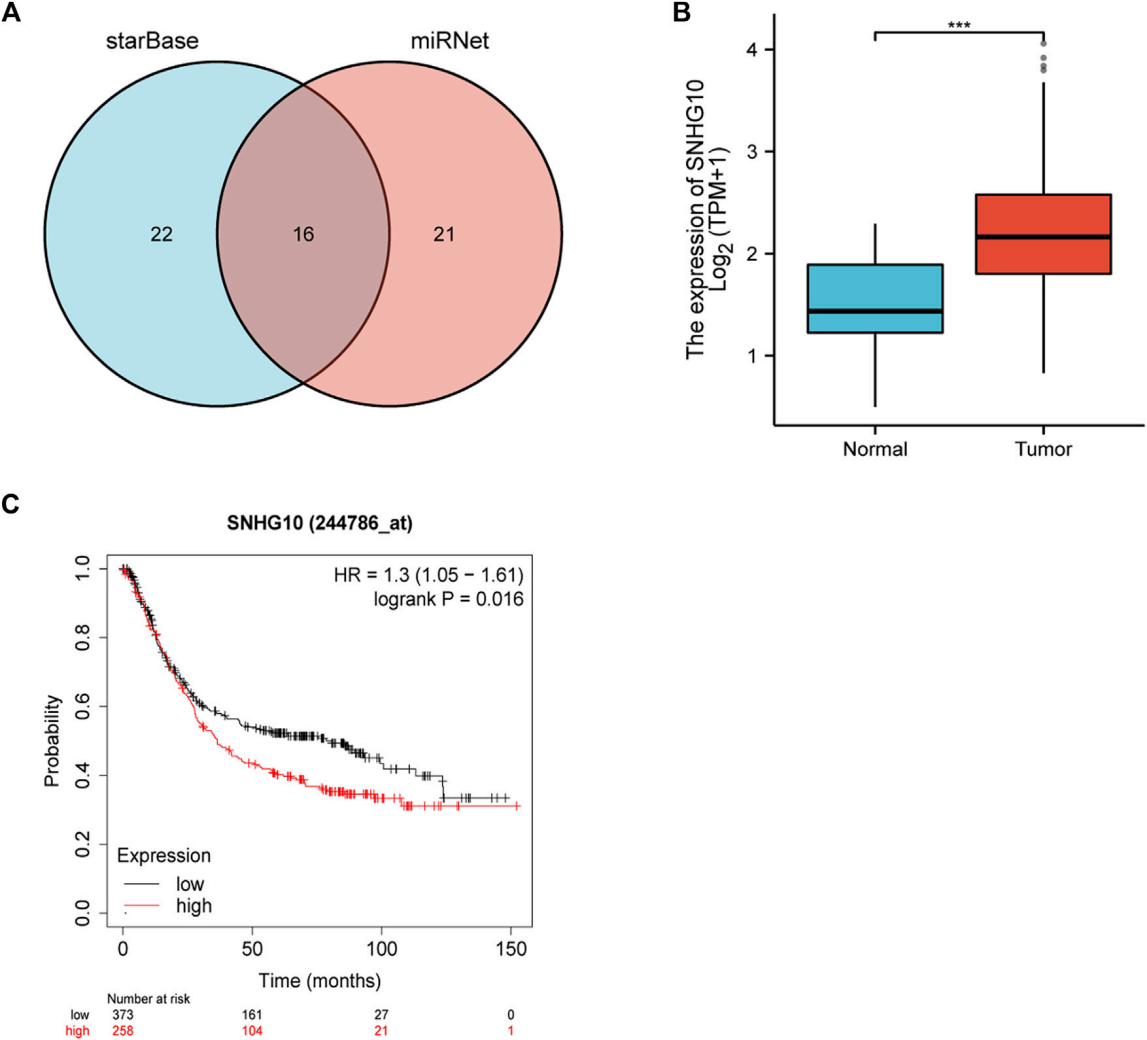
**(A)** Intersection of potential lncRNAs of hsa-miR-378-3p from starBase and miRNet databases. **(B)** Expression of SNHG10 in TCGA gastric cancer cohort. **(C)** Prognostic values (overall survival, OS) of SNHG10 in TCGA gastric cancer cohort.

**FIGURE 7:**
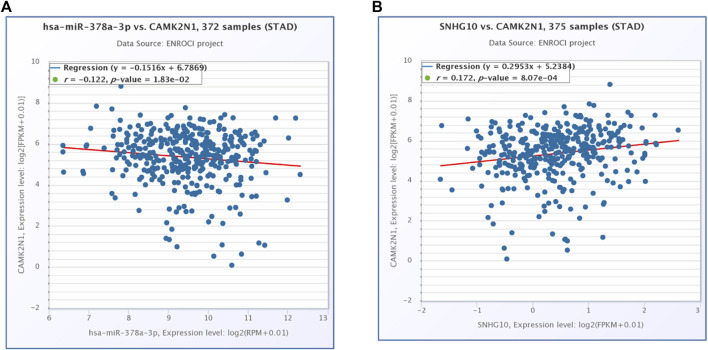
**(A)** Expression correlation of hsa-miR-378a-3p and CAMK2N1 in gastric cancer. **(B)** Expression correlation of CAMK2N1 and SNHG10 in gastric cancer.

The starBase database and miRNet database were utilized to predict the upstream potential lncRNAs that could potentially bind to the candidate miRNAs. As shown in Figure 6A, 16 lncRNAs were obtained as the ncRNAs that bind to hsa-miR-378a-3p. According to the competing endogenous RNA (ceRNA) hypothesis, lncRNA could increase mRNA expression by competitively binding to shared miRNAs. Therefore, lncRNA should be positively correlated with mRNA (Figure 7). The expression correlation between the four lncRNAs and miRNAs or mRNAs in GC was also determined by the starBase database. Then, we determined the expression levels and prognostic values of these lncRNAs in TCGA GC cohort (Figures 6B,C). Of all the predicted lncRNAs, only SNHG10 can meet all the conditions. Taking all expression analysis, survival analysis, and correlation analysis into consideration, SNHG10/hsa-miR-378a-3p/CAMK2N1 might be the most potential regulatory axis in GC.

### CAMK2N1 co-expression network in GC

The results of the co-expression pattern of CAMK2N1 are shown in Supplementary Figure S1A. Heat maps displayed the top 50 genes positively and the top 50 genes negatively associated with CAMK2N1 (Supplementary Figures S1B,C). GO term annotation showed that co-expressed genes of CAMK2N1 participate mainly in embryonic skeletal system development, epidermis development, isoprenoid metabolic process, organic cation transport, mesenchymal cell proliferation, modified amino acid transport, and columnar/cuboidal epithelial cell differentiation, etc. (Supplementary Figure S1D). The KEGG pathway analysis indicated enrichment in steroid hormone biosynthesis, glycerolipid metabolism, protein digestion and absorption, synaptic vesicle cycle, tight junction, and linoleic acid metabolism (Supplementary Figure S1E).

### Validation of the expression level of CAMK2N1, SNHG10, and hsa-miR-378a-3p in GC cell lines

**FIGURE 8:**
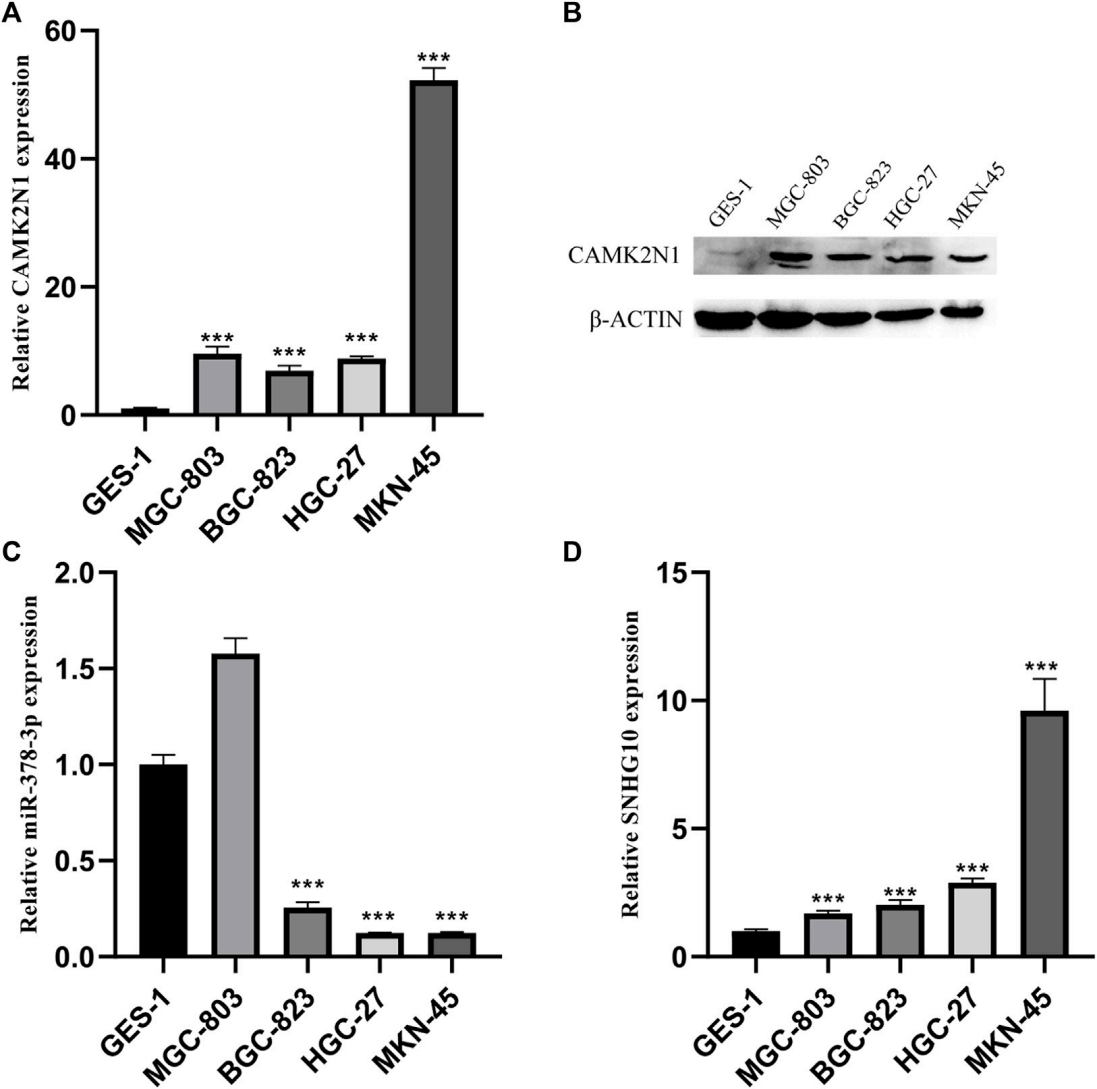
Expression levels of CAMK2N1, hsa-miR-378-3p, and SNHG10 in GC cell lines. **(A)** mRNA expression of CAMK2N1 in GC cell lines. **(B)** Protein expression of CAMK2N1 in GC cell lines. **(C)** Expression of hsa-miR-378a-3p in GC cell lines. **(D)** Expression of SNHG10 in GC cell lines.

To further validate the role of CAMK2N1, SNHG10, and hsa-miR-378a-3p in GC, we assessed the expression of mRNA and protein in gastric cancer cell lines. As shown in Figure 8, there revealed significantly over-expression of CAMK2N1 in four gastric cancer cell lines (MGC-803, BGC-823, HGC-27, and MKN-45) as compared to the normal gastric mucosal cell line GES-1 (Figures 8A,B). We found that SNHG10 was highly expressed in gastric cancer lines, and the miR-378a-3p was lowly expressed in BGC-823, HGC-27, and MKN-45.

### CAMK2N1 negatively correlates with immune cell infiltration in GC

**FIGURE 9:**
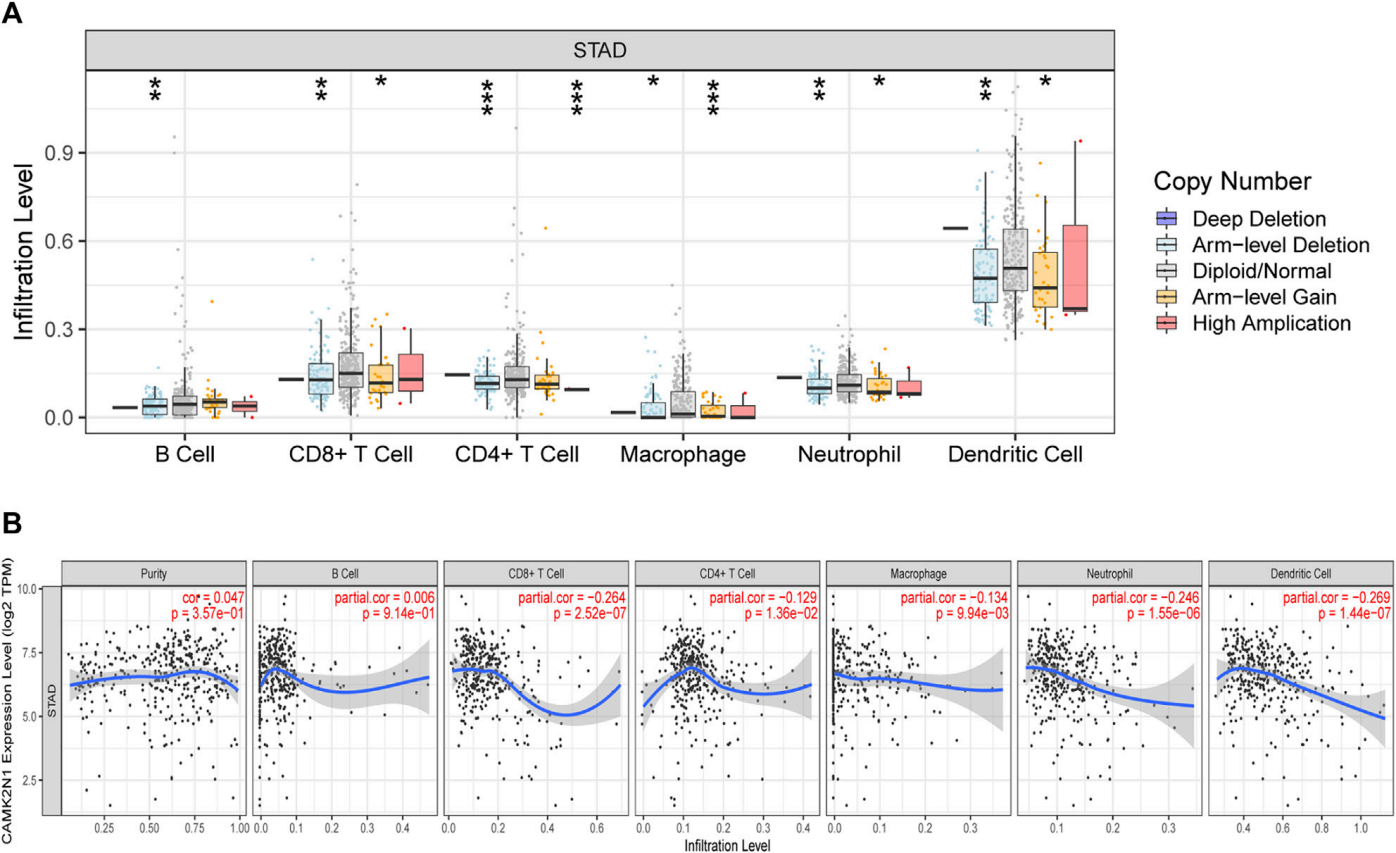
Relationship of immune cell infiltration with CAMK2N1 in gastric cancer. **(A)** Infiltration level of various immune cells under different copy numbers of CAMK2N1 in gastric cancer. **(B)** Correlation of the CAMK2N1 expression level with B cell, CD8^+^ T cell, CD4^+^ T cell, macrophage, neutrophil, or dendritic cell infiltration level in gastric cancer.

Calcium/calmodulin-dependent protein kinase II inhibitor alpha (CAMK2N1) is an endogenous cellular inhibitor of CAMK2. Recent studies revealed that the expression level of CAMK2N1 is also involved in the tumorigenesis of human cancers. As shown in Figure 9A, a significant change in immune cell infiltration level under various copy numbers of CAMK2N1 in GC was observed. Correlation analysis could provide key clues for studying the function and mechanism of CAMK2N1. Thus, the correlation of the CAMK2N1 expression level with the immune cell infiltration level was assessed. As shown in Figure 9B, CAMK2N1 expression was significantly negatively associated with all analyzed immune cells, including B cells, CD8^+^ T cells, CD4^+^ T cells, macrophages, neutrophils, and dendritic cells, in GC. This suggests that the overexpression of CAMK2N1 may be accompanied by the decline of immune cell infiltration in GC.

### Expression correlation of CAMK2N1 and biomarkers of immune cells in GC

To further explore the role of CAMK2N1 in tumor immune, we evaluated the expression correlation of CAMK2N1 with biomarkers of immune cells in GC using the GEPIA database. As listed in Table 3, CAMK2N1 was significantly negatively correlated with B-cell’s biomarkers (CD19 and CD79A), CD4^+^-T cell’s biomarkers (CD4), CD8^+^ T-cell’s biomarkers (CD8A and CD8B), M2 macrophage’s biomarkers (CD163, VSIG4, and MS4A4A), neutrophil’s biomarkers (ITGAM and CCR7), and dendritic cell biomarkers (HLA-DPB1, HLA-DRA, HLA-DPA1, CD1C, and ITGAX) in GC. These findings partly support the hypothesis that CAMK2N1 is negatively linked to immune cell infiltration.

**TABLE 3:** Correlation analysis between CAMK2N1 and biomarkers of immune cells in gastric cancer determined using the GEPIA database.

Immune cell	Biomarker	P	R
B cell	CD19	2.20E-04	−0.18
	CD79A	9.4E−08	−0.26
CD8^+^T cell	CD8A	1.5E−08	−0.28
	CD8B	1.5E−05	−0.21
CD4^+^T cell	CD4	2.5E−06	−0.23
M1 macrophage	NOS2	2.70E-01	0.055
	IRF5	4.60E-01	0.036
	PTGS2	3.10E-01	0.051
M2 macrophage	CD163	6.10E-04	−0.17
	VSIG4	1.20E-03	−0.16
	MS4A4A	2.6E−06	−0.23
Neutrophil	ITGAM	1.00E-03	−0.16
	CCR7	6.5E−09	−0.28
Dendritic cell	HLA-DPB1	6.6E−08	−0.26
	HLA-DQB1	5.90E-02	−0.094
	HLA-DRA	5.4E−07	−0.25
	CD1C	2.4E−06	−0.23
	NRP1	2.50E-01	−0.057
	ITGAX	6.90E-03	−0.13

### Correlation between CAMK2N1 and immune checkpoints in GC

**FIGURE 10:**
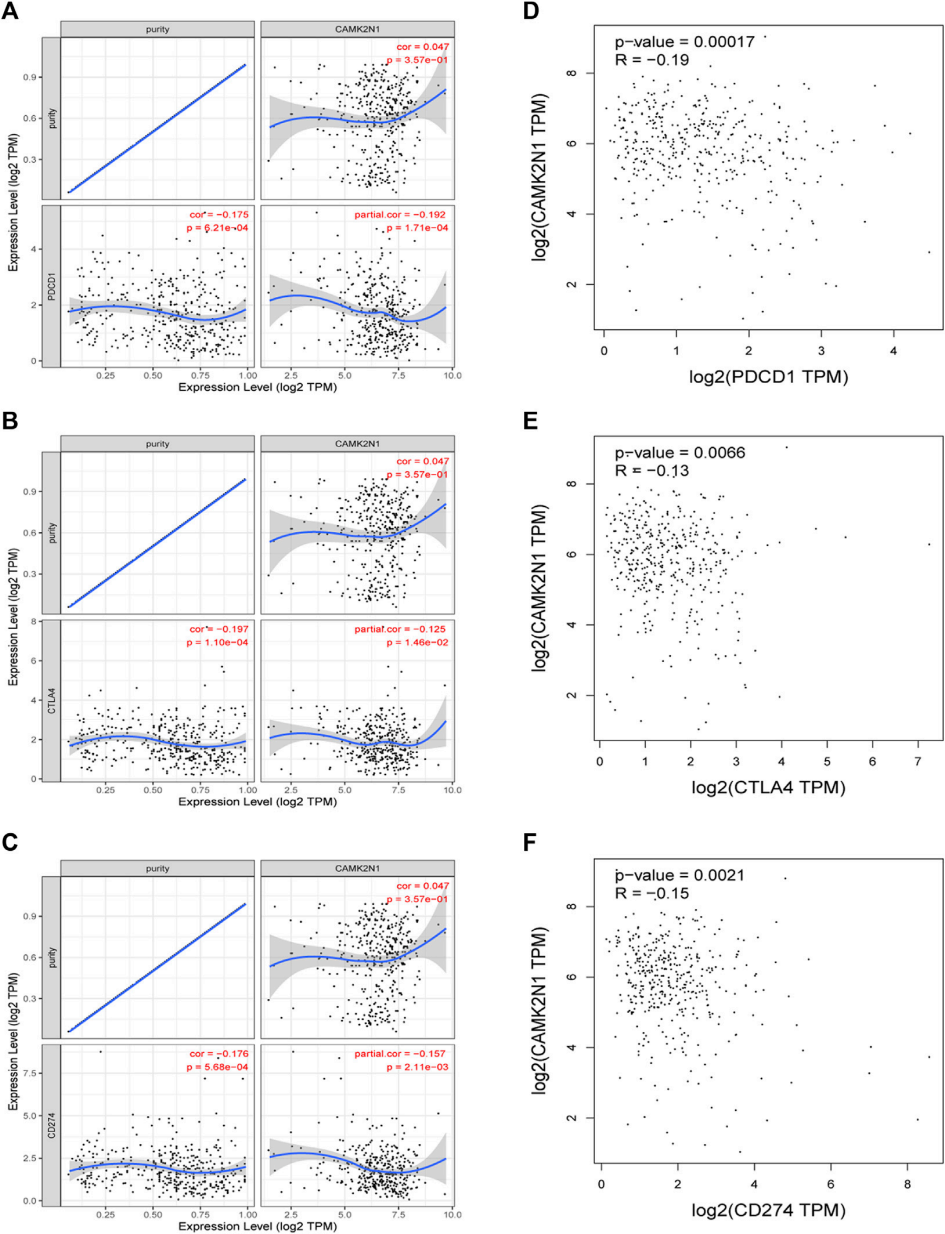
Correlation of CAMK2N1 with PDCD1, CTLA-4, and CD274 expression in gastric cancer. **(A)** Spearman correlation of CAMK2N1 with the expression of PDCD1 in gastric cancer adjusted by the TIMER database. **(B)** Spearman correlation of CAMK2N1 with the expression of CTLA-4 in gastric cancer adjusted by the TIMER database. **(C)** Spearman correlation of CAMK2N1 with the expression of CD274 in gastric cancer adjusted by the TIMER database. **(D)** Expression correlation of CAMK2N1 with PDCD1 in gastric cancer determined by the GEPIA database. **(E)** Expression correlation of CAMK2N1 with CTLA-4 in gastric cancer determined by the GEPIA database. **(F)** Expression correlation of CAMK2N1 with CD274 in gastric cancer determined by the GEPIA database.

PD1/PD-L1 and CTLA-4 are important immune checkpoints that are responsible for tumor immune escape. In order to explore the potential tumorigenic role of CAMK2N1 in GC, we assessed the relationship of CAMK2N1 with PD1, PD-L1, or CTLA-4. As shown in Figures 10A–C, CAMK2N1 expression was significantly negatively correlated with PD1, PD-L1, and CTLA-4 in GC, which was adjusted by purity. To further validate these results, we used the GEPIA database to examine the relationship between CAMK2N1 and immune checkpoints. We also found that there was a significant negative correlation of CAMK2N1 with PD1, PD-L1, or CTLA-4 in GC (Figures 10D–F). These results indicate that tumor immune escape might be involved in the CAMK2N1-mediated carcinogenesis of GC.

## Discussion

Until now, GC is still notorious for its poor prognosis and aggressive characteristics. Though great developments have been made in diagnosis and therapy for GC, the prognosis of patients is still perishing. Exploring novel molecular mechanisms of GC carcinogenesis may provide assistance to the development of effective therapeutic targets or seek valuable and helpful prognostic biomarkers. Increasing evidence has demonstrated that CAMK2N1 plays a key role in the proliferation and progression of multiple human cancers (Wang et al., 2014b; Li et al., 2018; Xu et al., 2019; Peng et al., 2021). However, the role of CAMK2N1 in GC is still unclear, and further studies are needed.

In this study, we first conducted comprehensive expression analysis and survival analysis based on TCGA cohort and GEO microarray. Then, seven key genes (CAMK2N1, OLFML2B, AKR7A3, CYP4X1, FMO5, MT1H, and MT1X) were identified which may be associated with the prognosis of GC. Furthermore, we established a ceRNA network based on seven key genes.

It has been widely reported that ncRNAs (miRNAs, lncRNAs, and circular RNAs) can play important roles in regulating biological behaviors by ceRNA mechanisms (Gao et al., 2020; Ghafouri-Fard et al., 2020; Razavi et al., 2021). We used the starBase database by seven prediction programs, involving PITA, RNA22, miRmap, microT, miRanda, PicTar, and TargetScan, to predict possible upstream regulatory miRNAs that could potentially bind to key genes. MiRNAs play a role in suppressing target genes by binding to the mRNA of target genes. Therefore, the miRNAs should be negatively related to mRNA, and eight miRNAs were finally obtained, including six miRNAs for CAMK2N1 and two miRNAs for FMO5. In addition, we take into consideration the prognostic significance and expression of predicted upstream regulatory miRNAs. According to the mechanism of miRNA, these miRNAs which bind to CAMK2N1 should be tumor-suppressive miRNAs, and these miRNAs which bind to FMO5 are tumor-promoting. We conducted this analysis in TCGA cohort using the Xiantao database and Kaplan–Meier plotter database. At the end, two miRNA–mRNA pairs (hsa-miR-378a-3p–CAMK2N1 and hsa-miR-34c-5p–FMO5) owned the most potential functions in GC and were selected for subsequent analysis.

According to the ceRNA hypothesis, the potential lncRNAs competitively binding to common miRNAs were found. The potential lncRNAs of hsa-miR-378a-3p–CAMK2N1 and hsa-miR-34c-5p-–FMO5 were first obtained by the starBase database. By conducting expression analysis, survival analysis, and correlation analysis, SNHG10 was found to be the most potential upregulated lncRNA for CAMK2N1. Also, we did not find suitable lncRNAs to construct a ceRNA network with FOM5. SNHG10 has been reported to function as an oncogene in multiple cancers, including GC. For instance, SNHG10 (Yuan et al., 2021; Zhang et al., 2021) enhanced cell proliferation and migration in GC. Taking together, SNHG10/hsa-miR-378a-3p/CAMK2N1 might be the most potential regulatory axis in GC.

CAMK2N1 is an endogenous cellular inhibitor of CAMK2 which is located on chromosomal 1 p1. Recent studies revealed that the expression level of CAMK2N1 is also involved in the tumorigenesis of human cancers. CAMK2N1 is commonly used as a tumor suppressor in some cancers, such as prostate cancer and hepatocellular carcinoma. It can inhibit E2F1 to regulate the cell cycle to affect the proliferation of hepatocellular carcinoma and regulate the proliferation of prostate cancer by suppressing ErbB2 (Wang et al., 2014b; Peng et al., 2021). But we found that CAMK2N1 may play a carcinogenic role in GC using qRT-PCR and Western blotting.

In order to verify the accuracy of the aforementioned bioinformatics analysis results, the expression of CAMK2N1, hsa-miR-378a-3p, and SNHG10 were verified in five GC cell lines (AGS, MGC-803, BGC-823, HGC-27, and MKN-45) by RT-qPCR and Western blotting. We found that the expression of CAMK2N1 and SNHG10 in GC cell lines were higher than that in the normal gastric mucosal cell line GES-1, and the expression of hsa-miR-378a-3p in GC cell lines was lower than that in GES-1.

A large number of studies (Li et al., 2016; Liu et al., 2017; Zhang et al., 2018; Dieci et al., 2021) have confirmed that tumor immune cell infiltration can affect the efficacy and prognosis of chemotherapy, radiotherapy, or immunotherapy in tumor patients. Our work indicated that CAMK2N1 was observably negatively correlated with various immune cells, including B cells, CD8^+^ T cells, CD4^+^ T cells, macrophages, neutrophils, and dendritic cells in GC. Moreover, CAMK2N1 was also notably negatively associated with biomarkers of these infiltrated immune cells. Our findings suggest that tumor immune infiltration may be partly responsible for the carcinogenic effect of CAMK2N1-mediated GC.

An immune checkpoint (Sharma and Allison, 2015; Wei et al., 2018; Kalbasi and Ribas, 2020) is a set of molecules expressed on immune cells that regulate the level of immune activation and play an important role in preventing autoimmunity (an abnormal immune function that attacks normal cells) from occurring. Abnormal expression and function of immune checkpoint molecules is one of the important reasons for the occurrence of many diseases, such as overexpression or over-function of immune checkpoint molecules, in which immune function is suppressed, the body’s immunity is low, and people are prone to cancer and other diseases. Conversely, if the immunosuppressive function of immune checkpoint molecules is poor, the immune function of the body will be abnormal. Efficacy of immunotherapy also depends on the sufficient expression of immune checkpoints in addition to adequate immune cell infiltration (de Miguel and Calvo, 2020; Morad et al., 2021). Therefore, we assessed the relationship between CAMK2N1 and immune checkpoints. The results demonstrated that high expression of CAMK2N1 was strongly linked to PD1, PD-L1, and CTLA-4 in GC, suggesting that targeting CAMK2N1 might increase the efficacy of immunotherapy in GC.

## Conclusion

**FIGURE 11:**
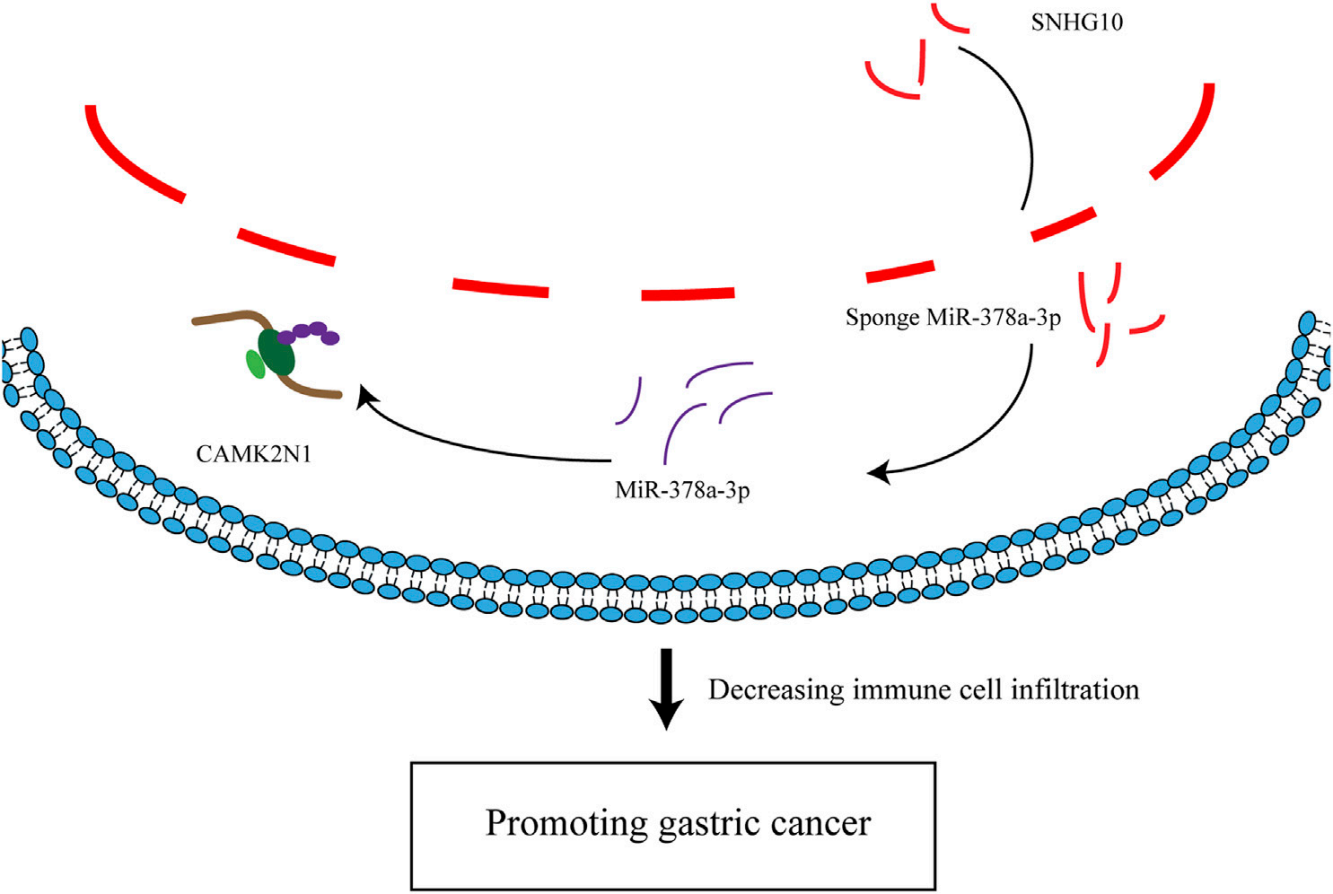
Model of the mechanism axis in carcinogenesis of GC.

In summary, we elucidated that CAMK2N1 was highly expressed in GC and positively correlated with unfavorable prognosis in GC. We predicted an upstream regulatory mechanism of CAMK2N1 in GC, which was the SNHG10/hsa-miR-378a-3p/CAMK2N1 axis (Figure 11). In addition, our current findings also indicated that CAMK2N1 might play its oncogenic roles through decreasing tumor immune cell infiltration and immune checkpoint expression. However, these results should be validated by many more basic experiments and large clinical trials in the future.

## Data Availability

The datasets presented in this study can be found in online repositories. The names of the repository/repositories and accession number(s) can be found in the article/Supplementary Material.
