# Supplementary figures and images for: NcRNA-mediated upregulation of CAMK2N1 is associated with poor prognosis and tumor immune infiltration of gastric cancer
Source: Front Genet. 2022 Aug 25;13:888672. doi: 10.3389/fgene.2022.888672 (PMC9452964; doi:10.3389/fgene.2022.888672)

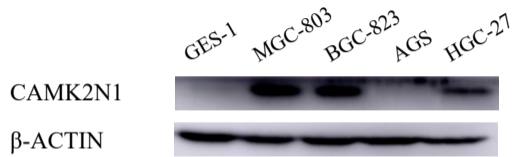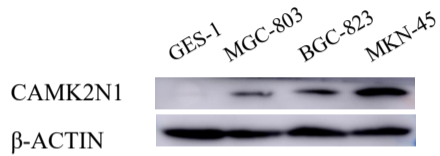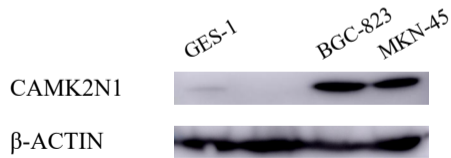

Supplement: Supplementary file 1 [file DataSheet2.PDF]

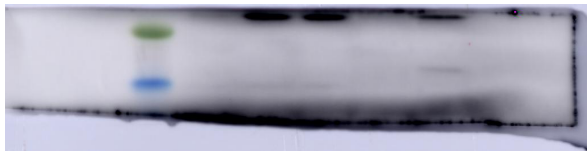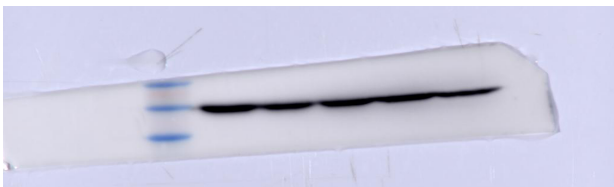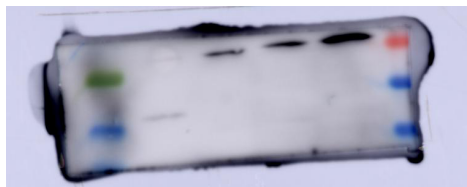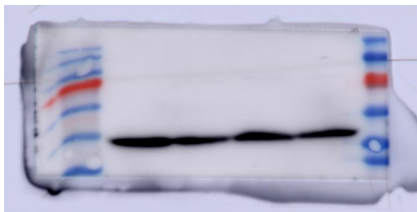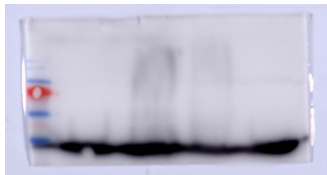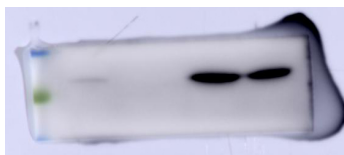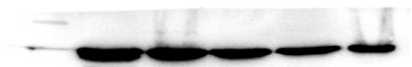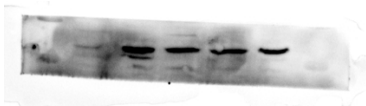

Supplement: Supplementary file 3 [file DataSheet3.PDF]

A

## CAMK2N1 Association Result

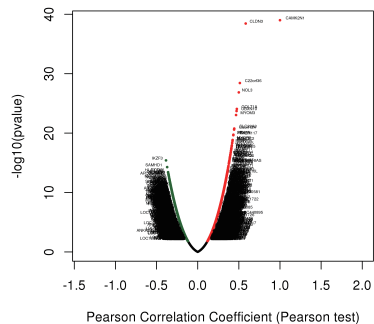

B

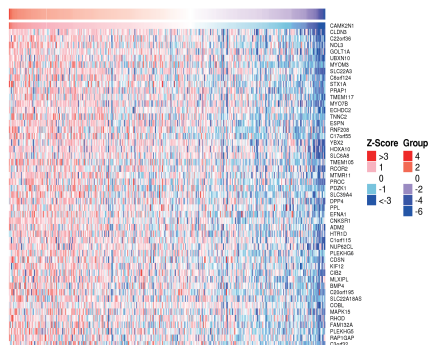

C

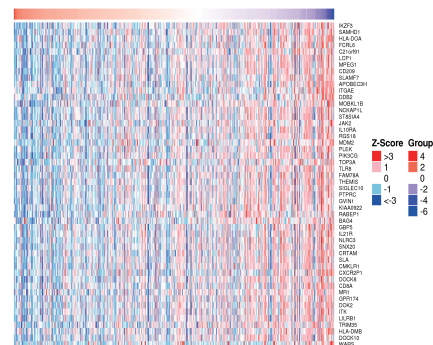

D

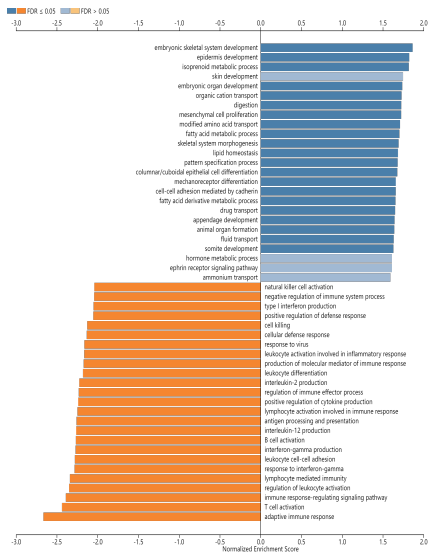

E

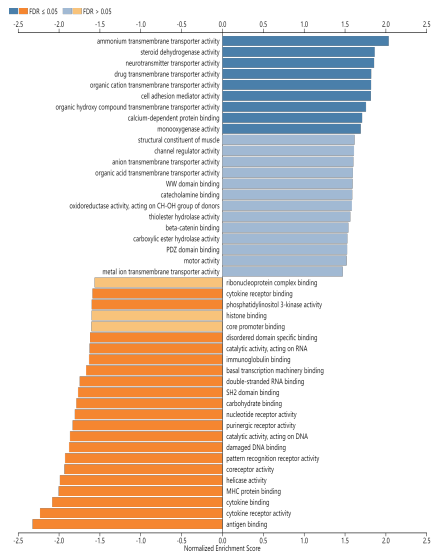

Supplement: Supplementary file 5 [file DataSheet1.PDF]
