# Supplementary material for: NcRNA-mediated upregulation of CAMK2N1 is associated with poor prognosis and tumor immune infiltration of gastric cancer
Source: Front Genet. 2022 Aug 25;13:888672. doi: 10.3389/fgene.2022.888672 (PMC9452964; doi:10.3389/fgene.2022.888672)
Supplement: Supplementary file 2 [file Table1.DOCX]

**Supplemental Table 1. Primary sequences used in this study.**

| **Gene** | **Primer Sequences (5’ to 3’)** |
| --- | --- |
| **CAMK2N1** | **FP: CGGAGCAAGCGGGTTGTTATT**  **RP: CGCCGTTCTTATTCTCTCCCT** |
| **GAPDH** | **FP: ACAACTTTGGTATCGTGGAAGG**  **RP: GCCATCACGCCACAGTTTC** |
| **Mir-378a-3p-RT** | **GTCGTATCCAGTGCAGGGTCCGAGGTATTCGCACTGGATACGACGCCTTC** |
| **U6-RT** | **CTCAACTGGTGTCGTGGAGTCGGCAATTCAGTTGAGAAAAATATGGAACGCT** |
| **U6** | **FP:** **CTGGTAGGGTGCTCGCTTCGGCAG**  **RP:** **CAACTGGTGTCGTGGAGTCGGC** |
| **Mir-378a-3p** | **FP: CGCGACTGGACTTGGAGTCA**  **RP: AGTGCAGGGTCCGAGGTATT** |
| **SNHG10** | **FP:** **CCAGCTTAGATTCATTGATTCC**  **RP: TTAAGTGCACCAGATGCTG** |
