# Supplementary material for: NcRNA-mediated upregulation of CAMK2N1 is associated with poor prognosis and tumor immune infiltration of gastric cancer
Source: Front Genet. 2022 Aug 25;13:888672. doi: 10.3389/fgene.2022.888672 (PMC9452964; doi:10.3389/fgene.2022.888672)
Supplement: Supplementary file 4 [file Table2.DOCX]

**Table S2.** Correlation analysis between SNHG10 and biomarkers of immune cells in gastric cancer determined by GEPIA database.

| Immune cell | Biomaker | P | R |
| --- | --- | --- | --- |
| B cell | CD19 | 6.0E-02 | -0.089 |
|  | CD79A | 1.5E-04 | -0.18 |
| CD8+T cell | CD8A | 7.6E-06 | -0.21 |
|  | CD8B | 8.8E-01 | 0.0074 |
| CD4+T cell | CD4 | 3.7E-08 | -0.26 |
| M1 macrophage | NOS2 | 8.7E-01 | 0.008 |
|  | IRF5 | 9.5E-04 | -0.16 |
|  | PTGS2 | 5.8E-01 | -0.027 |
| M2 macrophage | CD163 | 6.6E-03 | -0.13 |
|  | VSIG4 | 1.2E-03 | -0.15 |
|  | MS4A4A | 1.8E-04 | -0.18 |
| Neutrophil | ITGAM | 1.2E-04 | -0.18 |
|  | CCR7 | 3.6E-03 | -0.14 |
| Dendritic cell | HLA-DPB1 | 3.8E-08 | -0.26 |
|  | HLA-DQB1 | 5.2E-04 | -0.16 |
|  | HLA-DRA | 9.0E-05 | -0.18 |
|  | CD1C | 5.2E-05 | -0.19 |
|  | NRP1 | 5.6E-02 | -0.091 |
|  | ITGAX | 8.6E-04 | -0.16 |
